# Engaging rural communities in cancer prevention and control research: Development and preliminary insights from a community‐based research registry

**DOI:** 10.1002/cam4.4199

**Published:** 2021-10-14

**Authors:** Maria D. Thomson, April R. Williams, Arnethea L. Sutton, Katherine Y. Tossas, Charlotte Garrett, Vanessa B. Sheppard

**Affiliations:** ^1^ Department of Health Behavior and Policy School of Medicine Virginia Commonwealth University Richmond Virginia USA; ^2^ Office of Community Outreach and Engagement Massey Cancer Center Richmond Virginia USA

**Keywords:** cancer prevention, cancer risk factors, community outreach and engagement, community registry, health disparities, lifestyle behaviors, rural, screening

## Abstract

**Objective:**

To report on the development and preliminary findings of a community‐based cancer registry, including the community‐engaged approach to recruitment, participant profile, and distribution of cancer risk factors by race/ethnicity and geography.

**Methods:**

Community outreach and engagement best practices were used to recruit a diverse convenience sample of Virginia residents (≥18 years) that oversampled residents living in rural areas, defined as Rural‐Urban Continuum Codes (RUCC) 4–9 and African American (AA)/Black residents. Multiple survey administration methods included electronic (e‐survey) and in‐person survey by community‐based staff.

**Results:**

At the time of this analysis, 595 participants are enrolled; 73% are rural, 46% are AA/Black. AA/Black participants reported similar education but lower income (*p* < 0.01) and health literacy (*p* < 0.01), lower alcohol use (*p* < 0.001), fewer sedentary behaviors (*p* = 0.01), but greater BMI (*p* < 0.05) compared to White participants. Rural residents reported significantly lower household income (*p* < 0.001) and greater use of Medicaid (*p* = 0.01) compared to urban participants. Biennial mammography was reported by 82% of women aged 45–74 years old and colonoscopy by 77% of participants ≥50 years old. Tobacco use was reported by 17%; no differences in cancer screening or tobacco use were identified by geography or by race.

**Conclusion and relevance:**

Community engagement strategies successfully enrolled diverse residents within the cancer service area. AA/Black participants reported fewer cancer risk behaviors, similar educational attainment but lower income and health literacy compared to White respondents. Nuanced examinations of interactions among multilevel factors are needed to understand how individual, community, and institutional factors converge to maintain cancer disparities among AA/Black Virginians. Additional findings indicate a need for tobacco cessation, lung cancer screening, obesity treatment, and prevention initiatives.

## Graphical abstract

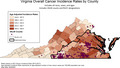

## 1 INTRODUCTION

The National Cancer Institute (NCI) cancer center program is the hallmark of cancer center research in the US, recognizing centers that achieve rigorous standards as NCI‐designated. Beginning in 2016, NCI‐designated centers have been mandated to establish formal community outreach and education^2^ (COE) efforts that address cancer burden within their catchment. To do this in a sustained way, resources are required that enable in‐depth examinations of structural elements within the sociocultural, environmental, economic, and institutional contexts of communities across the catchment area. NIH‐NCI has invested in cancer registries that are key for assessing incidence and mortality,^3^, ^4^ but underrepresentation of racial and ethnic minorities and rural communities continue to be a problem in cancer registries and research.^5^, ^6^ In response to this, a handful of community focused research registries have been established^7^, ^8^, ^9^, ^10^, ^11^ to truly reach and engage traditionally underrepresented community members who are less likely to participate in research efforts that utilize more traditional, arms‐length and random sampling recruitment procedures.^7^, ^8^, ^9^, ^10^, ^11^ COE activities have long been recognized as a critical complement to cancer disparities and health equity research.^1^

COE can facilitate the building of research capacity in historically underrepresented communities and foster bidirectional relationships that mutually set research priorities among researchers and community stakeholders.

The creation of the Virginia Living Well (VALW) community registry by the Massey Cancer Center (MCC) COE was driven by four overarching needs: (a) build research capacity in the community; (b) collect critical individual and family level data longitudinally to be linked to larger structural elements within communities; (c) build relationships with community stakeholders to improve access to clinical trials; (d) create mechanisms for community‐identified research priorities. In Virginia many of the predominantly rural counties located in the south central region of the state have significantly higher overall cancer mortality rates ranging from 168 to 246 per 100,000 compared to the state (161 per 100,000).^12^ Virginia is one of the few states that have been identified as having both breast and colorectal cancer hotspots, driven primarily by mortality rates among African American(AA)/Black Virginians.^13^, ^14^ While some community and state level data such as county health rankings are available (e.g., Robert Wood Johnson Foundation), more granular individual and family level data are required for tailoring programming, intervention, and clinical research. For example, in rural communities throughout Virginia there is limited knowledge about the interplay between individual level factors (health status, behavior, and attitudes) and community‐specific physical determinants (e.g., built environments, environmental exposures). The addition of these individual level data from racially and geographically diverse Virginians to existing community and state/policy level data would empower communities and researchers to work together to identify, develop and implement efficacious and lasting solutions to improve community infrastructure and healthcare access. This paper reports on the methodology used to build and recruit to the VALW, describes the community participant profile to date by race/ethnicity and geography, and presents preliminary insights about the distribution of known cancer risk/protective factors (access to care, cancer screening uptake, risk behaviors) and sociocultural and psychological factors known to influence access to health and cancer services.

## 2 DEVELOPMENT OF VIRGINIA LIVING WELL COMMUNITY REGISTRY

### 2.1 Outreach centers

MCC Office of Community Outreach and Engagement (COE) faculty and staff are located in one urban center and two rural communities (Lawrenceville and Danville) located within the cancer center catchment area. COE staff partner with a variety of community stakeholders to provide educational programming, connect residents with resources (e.g., transportation, navigation to cancer screening and provision of wigs for cancer survivors) and become trusted experts for cancer education resources located within their respective communities. Community stakeholders facilitate access into communities and are engaged with the registry through input on survey constructs and distribution methods. Leveraging existing community presence of two co‐located centers using the TRUST model, a set a characteristics that facilitate community‐engaged recruitment strategies, was a key engagement strategy^15^ and a natural extension for building research capacity in the communities served.^16^ Briefly, as advocated by the TRUST model, we designed an approach that included Trained multicultural and bilingual (English and Spanish) staff, interdisciplinary Researchers, Use of culturally relevant media, expanding social networks, community Spokespersons to facilitate community entrée, and development of culturally Tailored messages.^15^ To our knowledge, this is the first community‐based cancer prevention and control registry targeting rural communities in Virginia.

### 2.2 Team and research training of community‐based team members

Nine existing staff members who are residents of the communities in which centers are located, were trained to identify, recruit, obtain informed consent and administer the VALW survey. Most staff had no prior research training, thus completed the Collaborative Institutional Training Initiative (CITI Program) and several tailored modules covering topics including the history and protection of human subjects, ethical recruitment of participants, informed consent, survey administration, interviewing techniques, data entry and dissemination of research. Staff completed initial study‐specific trainings over a 4‐month period and maintain ongoing training through participation in a yearly, MCC Cancer Disparities Research symposium and periodic protocol refresher trainings.

### 2.3 VALW inclusion criteria

Eligible respondents are adult (age ≥18 years) residents of Virginia. Although not a criterion for inclusion, effort is made to oversample within rural communities throughout the cancer center catchment area. Rural is defined as rural‐urban continuum codes (RUCC) 4–9.^17^ Given the documented underrepresentation of rural populations in research studies,^18^ and the need for tailored rural cancer prevention and control programming, we leveraged our community partner network connected to the two COE offices located in rural VA counties.

#### 2.3.1 Consent

The survey was available as either an in‐person or an electronic (e‐survey). The e‐survey included a self‐guided e‐consent process. These modalities were developed following advice from community partners to address potential literacy or internet access barriers. The self‐administered e‐consent process uses embedded true/false questions to assess participant comprehension of key articles of consent. Incorrectly answered questions are followed by a prompt providing the correct information. In addition to the survey, participants are invited to consent to optional biospecimen (saliva) collection and optional participation in future research (agreement to join the registry). Individuals consenting to the registry agree to have their data stored indefinitely and will be invited to participate in additional research studies and cohort follow‐up surveys. Prior to roll out a certificate of confidentiality (COC) from the NIH and Institutional Review Board approval for human subjects research were obtained.

#### 2.3.2 Survey components

Choice of constructs was solicited from cancer prevention and control researchers and community stakeholders. Guided by best practices to minimize participant burden^19^ the objective was to collect data capable of facilitating conversations and action to address the catchment area cancer burden. Questions were adapted from national health surveys^20^, ^21^, ^22^ or commonly used scales.^24^, ^25^, ^26^ Demographics included race/ethnicity, attained education, annual household income, health insurance, and residential address. RUCC designations were assigned using zip codes from participants’ mailing addresses. Annual income was dichotomized using a threshold of $50,000. Though available in English and Spanish, this report is limited to English language survey responses.

*Cancer screening*. Participants were asked if they have ever participated in preventive screening for colorectal (age ≥50 years) or cervical (women, age ≥18 years) cancer. Mammography (women, age ≥40 years) was measured as participants who were screened ever and/or within the last 2 years.

*Cancer risk*/*protective behaviors* assessed tobacco use (yes/no), non‐medical drug use (yes/no), and time spent in sedentary activities (≥6 h per day). Excessive alcohol intake was determined for reports of ≥15 drinks or ≥8 drinks per week by men or women, respectively.^23^ BMI was calculated from self‐reported height and weight; ≥BMI 25 was considered overweight or obese.

*Access to health care*. Respondents were asked if they visited a primary care provider and dental provider in the last 12 months and if they heard of or participated in clinical trials.

*Sociocultural and psychological factors*: Structured scales were used to collect perceived discrimination (Everyday Discrimination Scale)^24^ and perceived stress (Perceived Stress Scale),^25^ where higher scores indicate higher perceived discrimination and stress, respectively. Depression was measured with a score of ≥16 on the Center for Epidemiological Studies Depression scale (CESD‐10).^26^ A one item health literacy screening question with established psychometrics was used.^27^, ^28^, ^29^

#### 2.3.3 Implementation and recruitment

To identify and recruit individuals in their community to the VALW, center staff used established relationships with local cancer coalitions, clinics, businesses, housing authorities, and government agencies such as social services to introduce VALW to community members. Posters and comment card boxes were distributed to key community partners to collect contact information.^16^ Events hosted by COE that included community lectures and educational programming were used to introduce and recruit for the project, as were community events hosted by partners (e.g., health fairs). These events were critical to reaching community members. Quick response (QR) codes embedded on advertising materials for distribution at large community events (e.g., health fairs, community lectures) enabled direct access to the survey without the need to be in‐person with center staff. Periodic updates were shared with community partners through community presentations and on study websites using slides, infographics, and short videos. Finally, to reach a broader audience radio, newspaper, and social media (Facebook) were used to distribute e‐survey invitations. VALW was launched in 2018.

#### 2.3.4 Statistical analyses

Descriptive statistics and tests for association (frequencies, chi‐square and *t*‐tests) were used to analyze baseline reports of access to care, cancer screening uptake, risk/protective behaviors and sociocultural and psychological factors among VALW respondents. Difference by race (AA/Black vs. White) and geography (rural vs. urban) were assessed.

## 3 PRELIMINARY FINDINGS

**FIGURE 1:**
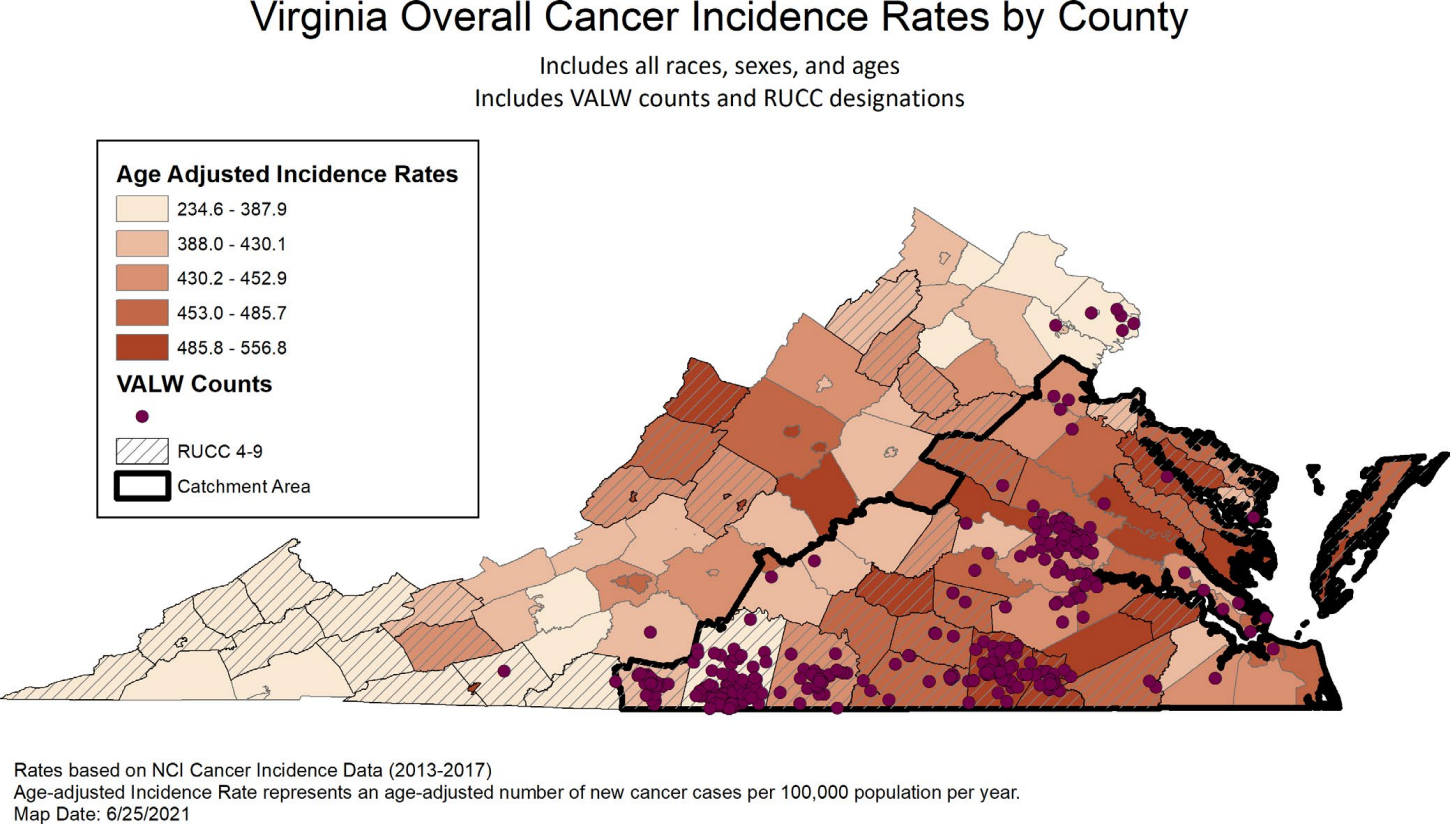
Virginia overall cancer incidence rates by county

**FIGURE 2:**
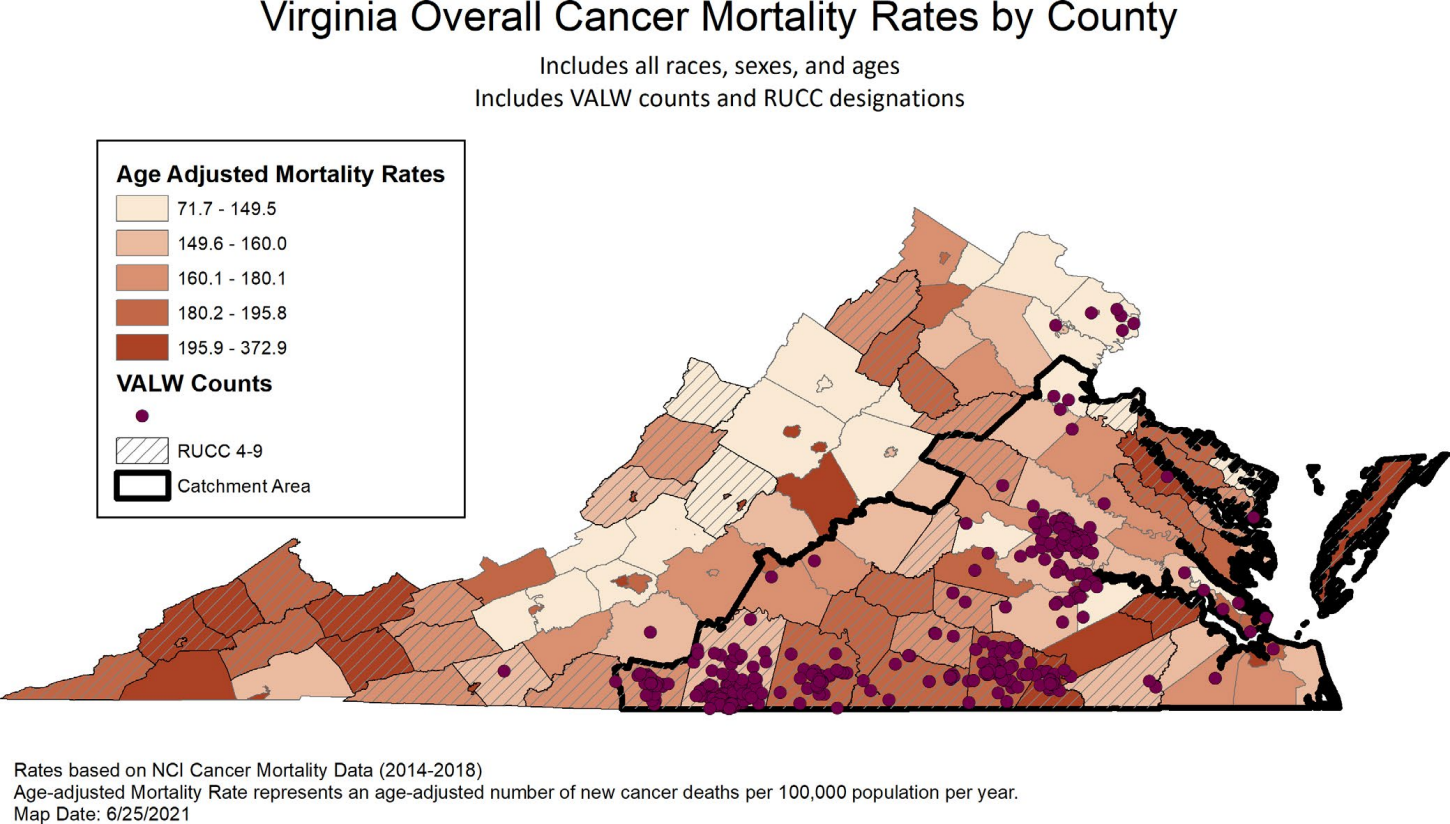
Virginia overall cancer mortality rates by county

Our community‐engaged model recruited 595 participants with high representation from historically underrepresented populations of rural Virginians (73% RUCC 4–9) and AA/Black Virginians (46%). Of the surveys completed, 57% used the in‐person, compared to the e‐survey. Figures 1 and 2 display the distribution of respondents within the catchment counties by rurality, cancer incidence, and mortality. The largest concentrations of respondents were centered on the three co‐located community offices; these figures show good representation of rural counties and counties with higher rates of cancer incidence and/or mortality. Overall, VALW participants were majority female (70%), with a median age of 52 years (range 18–85) and about half (52%) reported earning less than $50,000/household annually. About a third (39%) reported high school or less education and 7% reported having no health insurance (prior to COVID‐19), which mirrored Virginia state uninsured.^30^

Most had visited a doctor (92%) and a modest majority (65%) reported visiting a dentist in the last 12 months. High rates of ever cancer screening among age‐eligible adults were reported for breast cancer via mammography (91% women aged ≥40 years), cervical (85% women age ≥18 years), and colonoscopy (78% adults aged ≥50 years). Of the women age‐eligible for mammography screening, 82% reported having obtained a screen in the last 2 years. Many participants reported a BMI in the obese or overweight categories (71%), 17% of participants were current smokers, and 9% reported excessive drinking. Table 1 displays results by geography and race.

**TABLE 1:** Differences by race and geography N = 595

Variable	AA/Black n = 273 n(%)	White n = 282 n(%)	X^2^ [df] *p*	Urban n = 134 n(%)	Rural n = 443 n(%)	X^2^ [df] *p*
Saliva Biospecimen	138(52.1)	190(68)	**15.40[1] *p*** < **0.00**	95(75.4%)	244(56.1%)	**15.2[1] *p*** < **0.00**
Agree to be contacted	177(67)	222(80)	**12.50[1] *p*** < **0.00**	109(87.2%)	302(69.6%)	**15.5[1] *p*** < **0.00**
**Demographics**						
Unemployment	27(9.9)	19(6.7)	1.80[1] *p* = 0.17	17(13.0)	31(7.0)	**4.70[1] *p*** = **0.03**
Income < 50k	157(57.5)	133(47.2)	**5.91[1] *p*** = **0.01**	41(30.6)	256(57.8)	**30.45[1] *p*** = **0.00**
Private Insurance	130(47.6)	164(58.2)	**6.20[1] *p*** = **0.013**	81(60.4)	218(49.2)	**5.20[1] *p*** = **0.02**
Medicare	92(33.7)	78(27.7)	3.70[1] *p* = 0.054	31(23.1)	141(31.8)	3.72[1] *p* = 0.054
Medicaid	38(13.9)	34(12.1)	0.43[1] *p* = 0.514	11(8.2)	67(15.1)	**4.21[1] *p*** = **0.04**
Uninsured	20(7.3)	20(7.1)	0.01[1] *p* = 0.915	11(8.2)	31(7.0)	0.22[1] *p* = 0.63
Education: Highschool or less	117(43.7)	100(35.7)	3.61[1] *p* = 0.057	46(35.4)	179(41.4)	1.52[1] *p* = 0.21
**Access to Healthcare**						
Doctor last year	248(93.6)	251(89.6)	2.73[1] *p* = 0.098	117(90.7)	396(91.9)	0.18[1] *p* = 0.671
Dentist last year	157(59.5)	189(69.2)	**5.58[1] *p*** = **0.018**	83(68.0)	272(63.3)	0.94[1] *p* = 0.331
Heard of clinical trials	206(80.5)	243(90.3)	**10.30[1] *p*** = **0.001**	104(86.7)	355(84.7)	0.27[1] *p* = 0.598
Participated in clinical trials	29(14.1)	32(13.2)	0.08[2] *p* = 0.95	25(24.0)	37(10.5)	**13.71[2] *p*** = **0.001**
**Cancer Screening (ever screened)**						
Cervical Pap age ≥18	157(91.3)	185(92.5)	0.18[1] *p* = 0.66	82(96.5)	266(90.8)	2.92[1] *p* = 0.08
Mammogram age ≥40	120(96.8)	113(94.2)	0.96[1] *p* = 0.32	54(93.1)	183(96.3)	1.08[1] *p* = 0.29
Colonoscopy age ≥50	112(82.4)	114(88.4)	1.91[1] *p* = 0.16	50(89.3)	180(84.9)	0.70[1] *p* = 0.40
**Risk Behavior**						
Current smoking	46(18.3)	49(18.3)	0.00[1] *p* = 0.990	20(17.1)	78(18.8)	0.19[1] *p* = 0.66
Non‐medical drug use	11(4.2)	17(6.2)	2.57[2] *p* = 0.27	7(5.7)	19(4.4)	0.98[2] *p* = 0.61
Excessive drinking	13(4.8)	36(12.8)	**11.00[1] *p*** = **0.001**	14(10.4)	35(7.9)	0.86[1] *p* = 0.35
BMI>25	220(85.6)	174(64.4)	**31.20[1] *p*** = **0.00**	88(69.3)	314(75.3)	1.80[1] *p* = 0.1
Sedentary Risk	183(82.1)	210(89.7)	**5.59[1] *p*** = **0.01**	92(86.8)	314(86.0)	0.04[1] *p* = 0.84
**Sociocultural and Psychological Factors**						
Health Literacy	3.03(1.34)	3.25(1.12)	***t*** = **−2.09[509] *p*** = **0.037**	3.38(1.08)	3.05(1.29)	**2.80[225.9] *p*** = **0.006**
Depression (CESD)> = 16	31(12.2)	53(19.9)	**5.63[1] *p*** = **0.01**	21(17.1)	63(15.3)	0.22[1] *p* = 0.64
Perceived Stress (means SD/*t*‐tests; p)	5.60(SD 3.08)	6.09(SD 3.48)	*t* = −1.73[530] *p* = 0.08	5.70(SD 3.23)	5.86 (SD 3.33)	T = −0.46[205.7] *p* = 0.64
Everyday Discrimination (means SD/*t*‐tests; p)	5.46 (SD 5.53)	6.08 (SD 5.14)	*t* = −1.31[516] *p* = 0.18	6.03 (SD 4.76)	5.84 (SD 5.63)	T = 0.35[223.4] *p* = 0.72

^a^ Age missing = 30.

^b^ BMI missing = 35.

^c^ Sex missing = 9.

^d^ Race/ethnicity missing = 40.

^e^ Education missing = 16.

^f^ Income missing = 71.

^g^ Rural/urban missing = 18.

^h^ Insurance missing = 4.

### 3.1 Assessment of differences by geography

Participants living in rural areas reported higher use of Medicaid (15% vs. 8%; *p* = 0.04) and annual household income under $50,000 (58% vs. 31%; *p* < 0.001), but lower (pre‐COVID‐19) unemployment compared to urban participants (7% vs. 13%; *p* = 0.03). Despite no educational, access to care, or cancer screening differences, significantly fewer rural respondents reported participating in a clinical trial compared with urban respondents (11% vs. 24%; *p* = 0.001). Fewer rural residents agreed to provide biospecimen (56% vs. 75%; *p* < 0.000) or be recontacted for future research compared with urban residents (70% vs. 87%; *p* < 0.001).

### 3.2 Assessment of differences by race

No significant differences by race were identified in unemployment (pre‐COVID‐19), visiting a doctor, or education. Despite similar education, AA/Black respondents were more likely than Whites to report annual household incomes under $50,000 (58% vs. 47%; *p* = 0.015), were less likely to be privately insured (48% vs. 58%; *p* = 0.013), reported lower health literacy (*p* = 0.04), and were less likely to report a dental visit (60% vs. 69%; *p* = 0.02). Despite similar access to doctors and similar cancer screening uptake, fewer AA/Black respondents reported having heard about clinical trials (81% vs. 90%; *p* = 0.001), were less likely to consent to biospecimen collection (52% vs. 69%; *p* < 0.0001), or to agree to be recontacted for future research (67% vs. 80%; *p* < 0.001) compared with White respondents. Regarding obesity and other cancer risk behaviors, while AA/Black respondents were more likely to report higher BMI (86% vs. 64%; *p* < 0.001), they were less likely to report sedentary behaviors (82% vs. 90%; *p* = 0.018), depression (12% vs. 20%; *p* = 0.018), or excessive drinking compared with Whites (5% vs. 13%; *p* = 0.001). There were no racial differences identified by perceived stress or everyday discrimination.

## 4 DISCUSSION

Leveraging strong community partnerships, the VALW community registry has successfully recruited a racially and geographically diverse initial cohort, with enriched representation of AA/Black respondents (46% vs. 19% AA/Black population in VA) and those living in rural Virginia (RUCC 4–9) (73% vs. ~23% rural population in VA). The importance of a community‐engaged approach and physical presence in the community is evident by the high proportion of in‐person completed surveys, high rates of registry consent (71%), and moderate interest in biospecimen provision (59%). Several areas needing additional research or cancer programming were identified including tobacco cessation, obesity treatment and prevention, and clinical trial access.

Smoking was high in this sample (17% vs. 13.7% nationally),^31^ suggesting the need for concentrated outreach and engagement for tobacco cessation and lung cancer screening in the cancer center catchment area. The success of such programs will be influenced by purposeful and specific community, local clinic, and cancer center relationships^32^ and acknowledgment and understanding of relevant historical and contemporary factors related to tobacco production, marketing, sales, and use in Virginia. As such, continued use of community‐based approaches to program development, implementation, and evaluation are recommended.

Few differences were identified between rural and urban respondents. Similar to other studies,^18^ more rural respondents reported use of Medicare or Medicaid and annual household income under $50,000 and less access to clinical trials as compared with urban respondents. No other differences were identified in access to health care services, cancer screening behaviors, or cancer risk/protective behaviors for those living in rural compared with urban areas. These findings are in contrast to known determinants that affect access to clinicians and cancer screening services in rural areas.^18^, ^33^, ^34^ One explanation may be our use of a convenience sampling approach that leveraged existing community partnerships to identify and recruit rural residents. The community‐based offices have been in their respective communities since 2010 providing cancer education and programming.^16^ This initial round of VALW participants may over‐represent residents who have more salutary health behaviors (healthy user bias) and/or have previously been in contact with the centers, and thus have greater knowledge of available resources. However, given the underrepresentation of rural residents in cancer prevention and control research, it is critical to engage these residents in community‐based outreach and to understand community‐specific factors to better inform tailored interventions. Highlighted in these results are differences in clinical trial access. Rural and AA/Black respondents were more likely to report lower clinical trial access and to decline biospecimen and registry participation. Additionally, AA/Black participants reported lower health literacy and income as compared to Whites despite comparable education. These factors require more nuanced exploration as health literacy and income generally correlate with education. These may be reflective of historical and ongoing structural racism that impacts economic growth and access to research and clinical trials. While we did not measure medical or research mistrust, these have been linked to decreased participation among racial/ethnic minority and rural communities in research activities such as biospecimen collection and clinical trials.^39^, ^40^, ^41^

When stratified by race, AA/Black participants reported fewer cancer risk behaviors (i.e., lower alcohol use, greater mammography screening), similar smoking status, and higher overweight/obesity compared with White respondents. Given well‐documented links between several cancers and obesity^35^ and known disparities in cancer incidence and mortality among AA/Black and rural residents both nationally^13^, ^36^ and in Virginia,^14^, ^37^ a more nuanced examination stratified by geography and race/ethnicity that specifically examines multiple levels of influence (individual, interpersonal, and community) and domains of influence (behavioral, biological, physical environment, and sociocultural environment)^38^ are needed.

Key limitations include the convenience sampling and use of self‐report individual level data. Identification and recruitment methods likely influenced some findings pertaining to rural versus urban comparisons. Future efforts will focus on reaching rural and urban participants who have limited or no experience with the centers’ community outreach and education programming, as well as increasing representation of men and Latinx residents. Figures 1 and 2 display areas within the catchment that could be targeted for enhanced recruitment efforts. Finally, follow‐up with the initial cohort will be important to enable evaluation of temporal factors associated with cancer prevention and early detection behaviors.

## 5 CONCLUSION

To date, this work demonstrates a successful, community‐engaged strategy for recruitment of a sample that was enriched for AA/Black and rural Virginians, a critical step in the development of cancer prevention and control programming and research in rural Virginia. This initial sample identified key areas for cancer programming that included tobacco cessation, obesity treatment, prevention, and clinical trial access. Analyses that stratify these risks by important demographic variables of race/ethnicity, geography, and community level variables should next be compared with existing county, institutional, and state level data. As representation from different Virginia counties increases, the cohort established by this community registry can provide exciting opportunities to examine a broad range of structural and physical exposures within communities that may be influencing cancer risks.

## AUTHORS CONTRIBUTION

MDT and VBS conceived of the study; ARW and MDT completed the statistical analysis, CG lead data collection; MDT lead writing with co‐writers ALS, SRW, CG, KYT, and VBS.

## CONFLICT OF INTEREST

The authors declare that they have no conflict of interest.

## Data Availability

Precis: Community outreach and engagement builds bidirectional relationships among community members, patients, and researchers to prioritize community cancer needs. The Virginia Living Well Research and Registry (VALW) (currently n = 595) was developed to build bidirectional exchange relationships and collect critical longitudinal cancer prevention and control data within the cancer center catchment area.
